# Visitation patterns of endangered grey parrots (*Psittacus erithacus*) in a forest clearing in the Democratic Republic of the Congo

**DOI:** 10.1002/ece3.70039

**Published:** 2024-08-16

**Authors:** C. Fastré, B. Igwili, F. Van de Perre, Y. van der Hoek

**Affiliations:** ^1^ The Dian Fossey Gorilla Fund Musanze Rwanda

**Keywords:** behaviour, DR Congo, grey parrot, *Psittacus erithacus*, rainforest, temporal patterns

## Abstract

The grey parrot (*Psittacus erithacus*), once abundant, has become increasingly threatened due to the combined effects of capture for the global pet trade and habitat loss. Although grey parrots are well studied in captivity, effective conservation efforts require a better understanding of their ecological requirements in the wild. The aim of this paper is to quantify grey parrot behaviours across the annual cycle. To do that, we studied groups of grey parrots gathering in a natural forest clearing in the Nkuba Conservation Area, eastern Democratic Republic of the Congo. Using parrot counts and focal sampling, combined with descriptive statistics, we found that an average of 40 grey parrots visited the clearing each day, following a regular pattern in which they first perched in the trees surrounding the clearing, vocalizing loudly, until the group landed in the clearing to feed, drink and interact with each other. Generalized linear models (GLMs) and generalized additive models (GAMs) showed that the time at which parrots arrived, landed at, and left clearings was influenced by the weather, seasonality and the month of the year. We also found that parrots shortened their visits when disturbed by predators or the presence of humans. Although the underlying mechanisms for grey parrot visits to forest clearings remains unclear, the consistency of this behaviour observed elsewhere in Africa and the feeding observed in the clearing in this study suggest that these area support important foraging habitat for the wild grey parrots. Therefore, ensuring the availability of such clearings is paramount to the long‐term survival of the species. We suggest that future efforts to protect grey parrots in their native habitats focus on identifying clearings visited by parrots, monitoring these clearings and allowing parrots to visit them without disturbance or risk of capture.

## Graphical abstract

We observed endangered grey parrots visiting a natural clearing in the rainforest of the Nkuba Conservation Area in DR Congo. We found that the frequency and duration of these visits vary with the weather, the season and disturbances such as human activity. Visiting such clearings provides parrots with important food resources and constitute an important part of their life‐cycles. Such clearings should be identified across their range and protected.

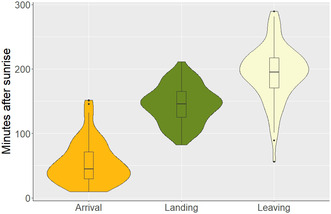

## 1 INTRODUCTION

The endangered grey parrot (*Psittacus Erithacus*) is a once‐abundant keystone species (Blanco et al., 2018) in its 5‐million‐hectare range throughout west and central Africa (BirdLife International, 2022; IUCN, 2023). Most previous studies on wild grey parrots have examined their habitat‐use, their diet and threats to the species in its African range (Amuno et al., 2007; Annorbah et al., 2015; Chapman et al., 1993; Chupezi et al., 2006; Dueker et al., 2020; Hart et al., 2016; Marsden et al., 2016; May, 2001; Piebeng et al., 2018; Tamungang & Ajayi, 2003; Tamungang et al., 2014, 2016). These studies have mainly found that wild grey parrots inhabit both primary and secondary rainforests where they feed on a large array of plant parts such as seeds, fruits, leaves and buds. Grey parrots roost in groups and their breeding season lasts from March to November when they raise an average of two chicks in tree cavities. Grey parrots are prey to several animal species such as cats and raptors.

Unfortunately, grey parrot populations underwent rapid declines in the last decades (50–79%, IUCN, 2023) due to a combination of factors including capture for the pet trade (Annorbah et al., 2015; Marsden et al., 2016; Martin, 2018) and deforestation (Hart, 2013; IUCN, 2023; Martin et al., 2014). Although these declines have drawn the attention of the conservation community (Amuno et al., 2010; Annorbah et al., 2015; Chapman et al., 1993), few studies have attempted to close the knowledge gap on behaviours of grey parrots in the wild (Bentley, 1999; Hart, 2013; May, 1996), although such knowledge is crucial for the protection of the species, and a priority for future research (Martin et al., 2014).

It is challenging to study wild grey parrots as they live in remote forests, travel daily across large distances and are difficult to observe while feeding in high canopies (Amuno et al., 2007; Chapman et al., 1993; Marsden et al., 2016; Martin et al., 2014). Instead, grey parrots may be studied at communal roosts, at drinking sites or when visiting natural forest clearings characterized by marshy or muddy soil (Martin et al., 2014; Tamungang et al., 2014). Such clearings—also referred to as salt/clay/mineral clearings/licks—attract large numbers of parrots and are likely to play a key role in parrot ecology as they contain minerals important for the parrots' diet (Chupezi et al., 2006; Hart, 2013; Martin et al., 2014). Geophagy is common among parrots (Burger & Gochfeld, 2003) and is often reported in South America where various Neotropical parrots visit clay licks to ingest clay and supplement or detoxify their diet (Brightsmith & Villalobos, 2011; Burger & Gochfeld, 2003). Unfortunately, because these clearings attract large flocks of grey parrots that are vulnerable when foraging on the ground, they are sought after hunting grounds for poachers (Hart, 2013).

Forest clearings visited by parrots are likely scarce in the Congo basin (Hart, 2013) and few such clearings have been reported in literature (Table 1). In each case, flocks of hundreds of grey parrots were seen foraging and drinking on the ground. Studies on the behaviour of grey parrots in clearings in Lobéké and Dzanga NP (Bentley, 1999; May, 1996, 2001), carried out from a hide every morning, showed that parrots started gathering in the trees surrounding the clearing an hour after dawn, after which the first parrots descended into the swamp to forage on plants and soil (Bentley, 1999). As the reported observations were limited to a single season (dry season in May, 1996 and Bentley, 1999) or even single observations (Hart, 2013), there is so far no knowledge on how parrots use clearings throughout the year.

**TABLE 1:** Overview of studies on grey parrots in salt licks in chronological order.

Reference	Country	Location (name of the clearing)	Year of study	Study period	Number of parrots reported	Trapping reported
May (1996)	Central African Republic	Dzanga‐Sangha Reserve	1995	Mid June to mid‐August	30–300	No
Bentley (1999) May (2001)	Cameroon	Lobéké Reserve (Boulou Savanne)	1997	Late June to mid‐August	200–800, 800+	Yes
Hart (2013)	Democratic Republic of Congo	Okapi Reserve (Mehwa)	2005	NA	Hundreds	No
Hart (2013)	Democratic Republic of Congo	Lomami National Park (Parc des Perroquets)	2011	March (unique observation)	NA	Yes
This study	Democratic Republic of Congo	Nkuba Conservation Area (Ungwe)	2021	July 2021–July 2022	155	No

Yet, several factors may influence the visitation patterns of the clearings by grey parrots, including rainfall seasonality. Wet and dry seasons are characterized by marked differences in food availability within the forest, especially a lower amount of fruits during the dry season (Adamescu et al., 2018; Tamungang & Ajayi, 2003; Van Der Hoek et al., 2021), and would therefore influence how much parrots would rely on the resources found in the clearing. The breeding season of the parrots, which lasts from March to November, may also impact the use of clearings by parrots as they provision young (mainly during the dry season of June–September, Piebeng et al., 2018). Studies carried out in licks in the Amazon indeed show that parrots visit licks most often during the breeding season when they need additional nutrients (Brightsmith et al., 2018). Finally, potential disturbances, like the presence of predators or humans, could cause the parrots to leave the area earlier than expected (May, 1996). Given the importance of clearings for the grey parrot and the significant threat of poaching, more information on visitation patterns throughout the year is urgently needed.

Here, we report the visitation patterns of grey parrots in a clearing in the easternmost part of their range: the Nkuba Conservation Area (NCA) in eastern Democratic Republic of the Congo (DRC). The aim of our study is to quantify grey parrot behaviours across the annual cycle as they visit the clearing. We first tested whether visitation timings (arrival, landing and departure times), visit duration, visitation rate (presence and absence of the parrots) and the group size of the parrots are consistent throughout the year or varies among seasons, as food availability in the forest can vary between the wet and the dry season, and therefore, influence the visitation patterns and the number of parrots in the clearing. Second, we tested whether morning weather, which was shown to affect visitation patterns of parrots in licks in South America (Brightsmith, 2004), influenced visitation patterns and group size. Third, since the occurrence of disturbances could influence departure time and visit duration, we included disturbance in the model for those two response variables. Second, we tested whether activities of the parrots in the clearing (feeding, drinking, preening, interacting, moving around the clearing or mating) differed between seasons as the need for food or water from the clearing or the occurrence of mating activities may be dependent on the time of the year. Lastly, we discuss the implications of our findings for conservation actions.

## 2 MATERIALS AND METHODS

### 2.1 Study site

**FIGURE 1:**
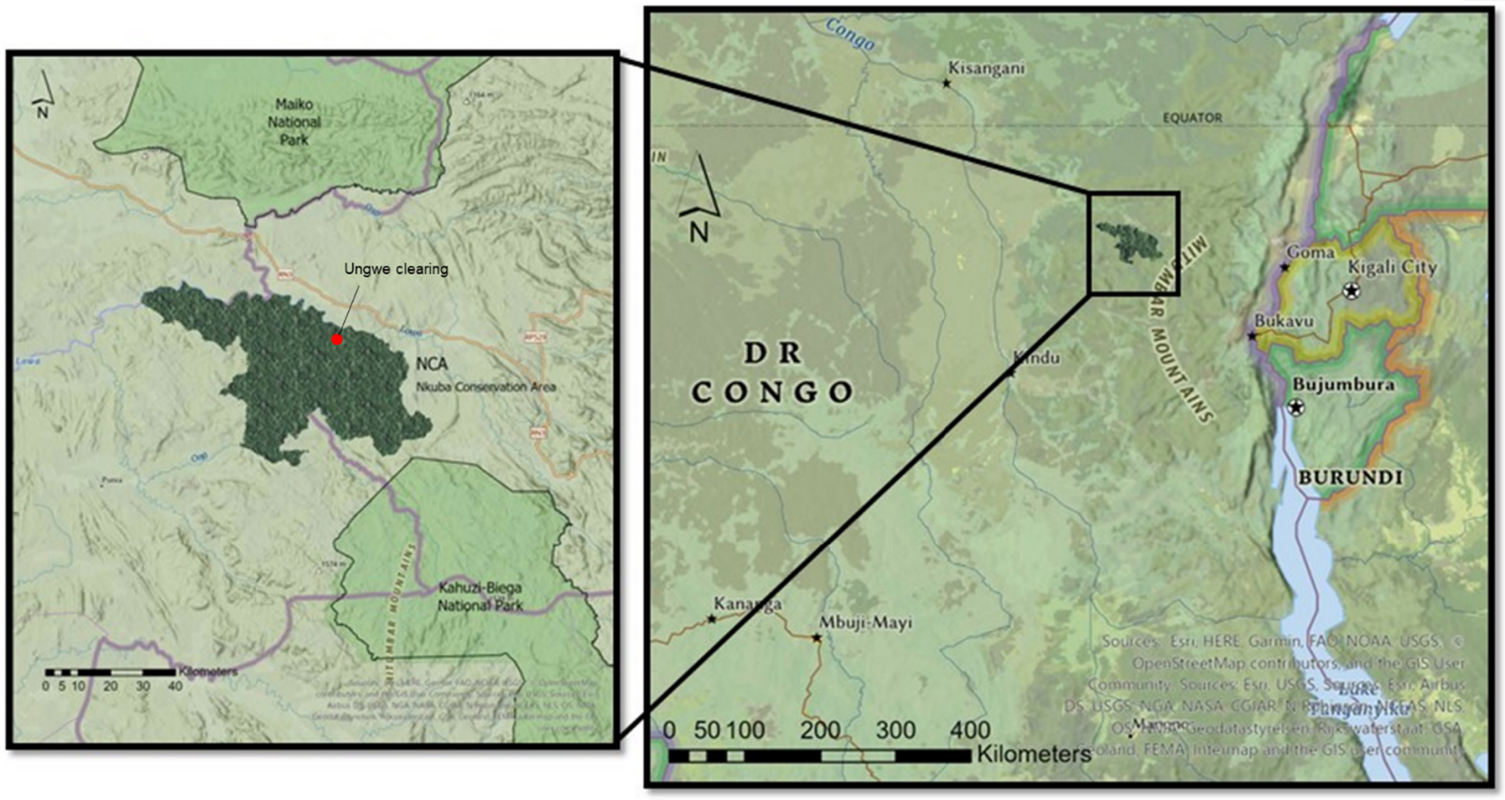
The Nkuba conservation area (NCA) located South of Maniema National Park and North of the Kahuzi‐Biega National Park in eastern Democratic Republic of the Congo. In red is the location of the Ungwe clearing.

The focal forest clearing is found in the Nkuba Conservation Area (hereafter NCA; 1°17′ S, 27°31′  E; ~500–1500 m.a.s.l.), a ~2460 km^2^‐large community reserve located in the North Kivu and Maniema Provinces, DRC. The NCA is located between Maiko and Kahuzi‐Biega NP (Figure 1). The area is covered by lowland rainforest and has a tropical climate with average annual temperatures of 24.2°C and precipitation between 2100 and 2500 mm yr^−1^, with peaks during two wet seasons from April to May and September to December (Karger et al., 2017). Conservation and ecological research activities in the NCA were initiated in 2012 by the Dian Fossey Gorilla Fund (DFGF), which still manages conservation actions and research in the area in cooperation with local communities.

**FIGURE 2:**
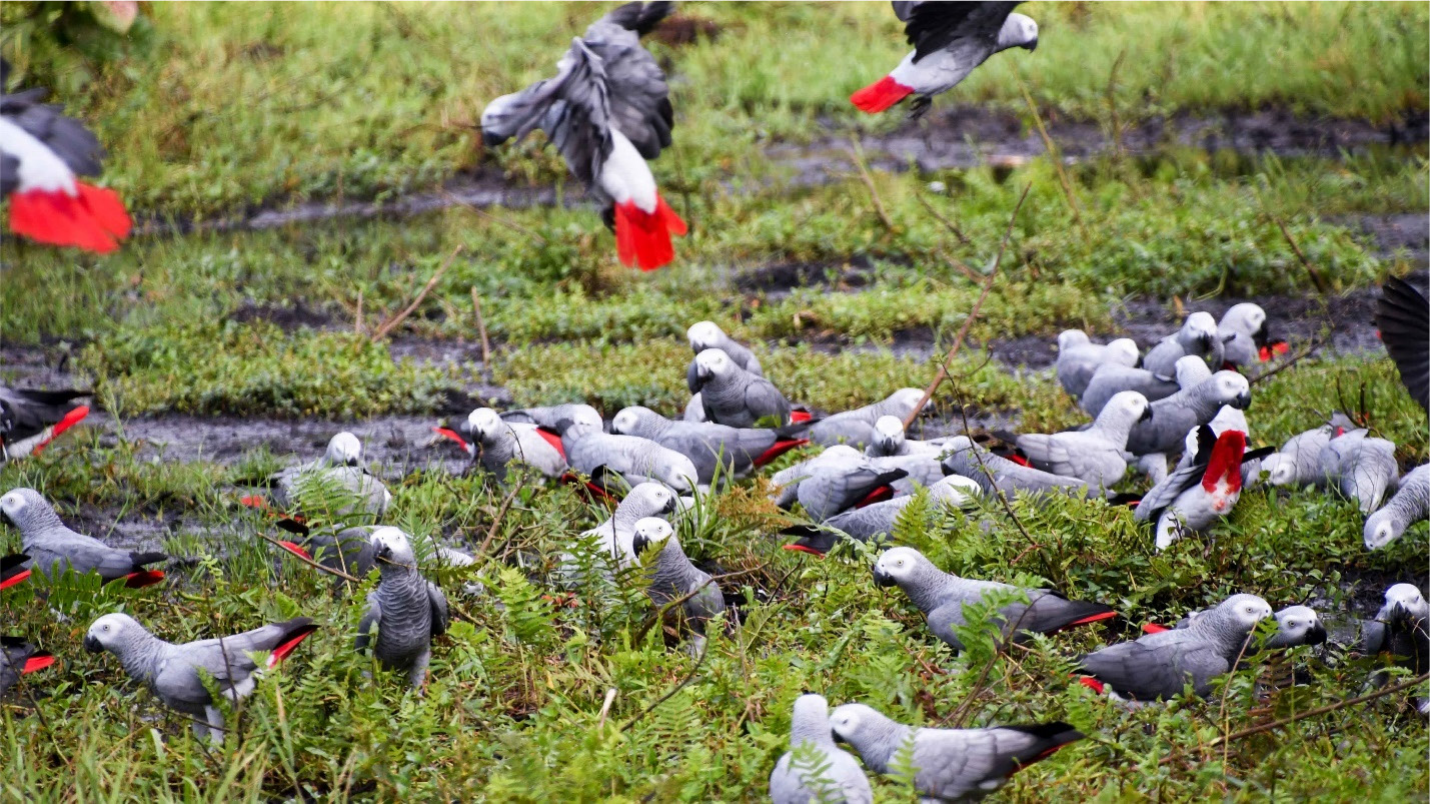
Picture of a flock of grey parrots visiting the Ungwe clearing in the Nkuba conservation area in Democratic Republic of Congo (author C. Fastré).

The NCA harbours several forest clearings, but only one (locals call ‘Ungwe’) was regularly visited by groups of grey parrots (Figure 2) according to advice from local community members and exploratory efforts from the DFGF team. The clearing includes a variety of tall grasses, various water plants and stagnant water, and is surrounded by large shrubs and deciduous trees. Although the NCA was recently designated a community reserve, pigeon trappers are established in the area around the clearing and use glue traps in treetops to capture green pigeons. This clearing is visited by many vertebrate species including African forest buffalo (*Syncerus caffer nanus*) and Grauer's Gorilla (*Gorilla beringei graueri*; Personal observations, Table A1). African forest elephant (*Loxodonta cyclotis*) is currently absent from the NCA.

### 2.2 Data collection

Between July 2021 and July 2022, two trained observers, including B.I., visited Ungwe for nine periods of 7–12 consecutive days spread over the year and across seasons (for a total of 276 h of observation across 100 days). However, due to logistical constraints (lack of available trained observers and access to the clearing made difficult by the heavy rains), we were unable to visit the clearing during the short rainy season of April to May and during the month of September. Each observation session started at approximately 6 a.m. before the parrots had arrived in the area and ended before noon when all parrots had left the area. During this time, observers stayed concealed in trees surrounding the clearing. To answer the abovementioned research questions, we collected five types of information on the parrots' visits to the clearing: timings, group size, behaviour, disturbances and environmental variables.

Observers recorded the ‘arrival time’, the time at which the first parrot perched in trees adjacent to the clearing, often detected as they vocalized, and the ‘landing time’, the time at which the first parrot landed on the ground in the clearing. The session ended when all parrots had departed from the area, which was recorded as the ‘departure time’. These times were subsequently transposed to minutes after sunrise to account for variation in day length throughout the year (See Table A2 for sunrise hours in the study area). From these, we calculated two additional metrics: the ‘presence/absence’ of the parrots on any given observation day in the clearing as whether they landed on the ground of the clearing (presence) or not (absence) and the ‘visit duration’ as the amount of time between the landing of the first parrot and the departure of the last parrot from the clearing.

We determined the daily ‘group size’, which we considered as the maximum number of parrots that landed in the clearing during an observation session. Group size was determined based on the counts of the number of parrots present in the group located inside the clearing performed every 10 min from landing time to departure time. It was impossible for the observers to evaluate how many parrots were perched in the trees from their observation point situated inside the forest, and we must therefore use the maximum number of landed parrots as a proxy for the total group size for each session.

In between these counts, the observers performed focal sampling of the behaviour of individual parrots (Martin & Bateson, 2007). Based on the focal sampling data, we calculated the percentage of time focal animals spent on predefined behaviours. In focal sampling, the duration of all the behaviours (moving, drinking, feeding, mating, preening or interacting) of a randomly selected parrot was continuously recorded over the course of 5 min or until the individual was out of sight. If the focal individual was out of sight within the first 2 min of the sampling, the observers selected another individual. They followed this bird for 5 min or until this individual was out of sight, until the next parrot count. Note that, when parrots were foraging on food items on the ground, it was impossible for the observers to identify with certainty whether they fed on soil or plants. Even when parrots pulled plants out of the ground to consume them, observers were unable to distinguish which part of the plant or which species of plant was consumed.

In addition to visitation data or patterns, we recorded environmental variables that would be relevant to explain visitation patterns. First, the observers recorded three types of disturbances—human noises, the presence of raptors, and alarm calls emitted by great blue turacos (*Corythaeola cristata*) — as these could scare off the parrots and influence the departure time and therefore the duration of their stay (May, 1996). Second, we registered whether it rained in the morning during the observations (morning weather) as rain was shown to influence visitation rates of parrots in licks in Peru (Brightsmith, 2004). Third, observation dates were classified into wet and dry season based on the month (i.e. wet from April to May and September to December).

We ran Pearson's Chi‐squared tests between morning weather, season, disturbance and group size to see if they were correlated and evaluated the strength of the relationship of correlated variables using a Cramer‐V test, using the ‘tidyverse’ and ‘lsr’ packages in R (Navarro, 2015; R Core Team, 2022; Wickham et al., 2019). Cramer's V falls between 0 and 1 (where 0 indicates no association and 1 would indicate a perfect association). We considered a value of >0.15 to be a strong association (Akoglu, 2018) and refrained from using explanatory variables in the same model if their Cramer's V  was above the 0.15 threshold. We found no significant correlation between season and disturbance (X^2^ *p*‐value = .02). Morning weather was moderately correlated to both season (there are more rainy mornings during the wet season, X^2^ *p*‐value = .12 and Cramer V = 0.12) and disturbances (there are less disturbances on rainy mornings X^2^ *p*‐value = .38 and Cramer V = 0.13). Group size was strongly correlated to all three environmental variables (Season X^2^ *p*‐value = .65 and Cramer V = 0.66, Morning weather X^2^ *p*‐value = .50 and Cramer V = 0.50 and Disturbances X^2^ *p*‐value = .1 and Cramer V = 0.78). We therefore did not use group size as an explanatory variable.

### 2.3 Statistical analyses

We first described the visitation patterns of daily visits by reporting the maximum, minimum, mean and standard deviation (SD) of arrival time, landing time, departure time and visit duration. As arrival time was not normally distributed (Shapiro test, *p*‐value <.001), we added the median and 1st and 3rd quartiles. Because daily activities of the parrots may be affected by seasonal food or water availability, we tested whether the time spent on behavioural activities during a given visit varied between dry and wet seasons using linear models from the stats package (R Core Team, 2022). We then fitted generalized linear models (GLMs), which do not assume data normality, to determine whether season (wet or dry) or morning weather influenced any of the response variables (visit duration, arrival time, landing time, departure time, presence/absence and group size), and whether disturbance influenced departure time and visit duration. We used the glm function of the ‘stats’ package in R (R Core Team, 2022) with Gaussian or logistic (for presence/absence) distributions. We considered *p*‐values of less than .05 significant. We consider models with *R* ^2^ values of at least .1 to be biologically meaningful while results from models with *R* ^2^ smaller than .1 should be interpreted with caution (Chalmer, 1987).

Apart from differences among seasons, we looked for ‘within‐season’ fluctuations in visitation patterns by determining for which month visitation patterns and group size were lowest and highest. While fully acknowledging the limitations of our data (e.g. we lacked data for September, March, April, and May), we also fitted generalized additive models (GAMs) with months fitted along a smoothing spline (knots = 9) as a predictor variable for presence/absence of parrots (binomial), visit duration, group size, arrival, landing and departure times (all Gaussian). Given the scarcity of our data overall as we lacked data from 4 months, and the even more limited number of observations of specific activities (e.g. drinking), we opted not to use GAMs to examine temporal patterns in observed behaviours. We used R packages ‘mgcv’ (Wood & Wood, 2015) and ‘MASS’ (Ripley et al., 2013) to fit these models and derived fitted values using the ‘itsadug’ package (van Rij et al., 2020). All statistics were performed in R (R Core Team, 2022).

## 3 RESULTS

**FIGURE 3:**
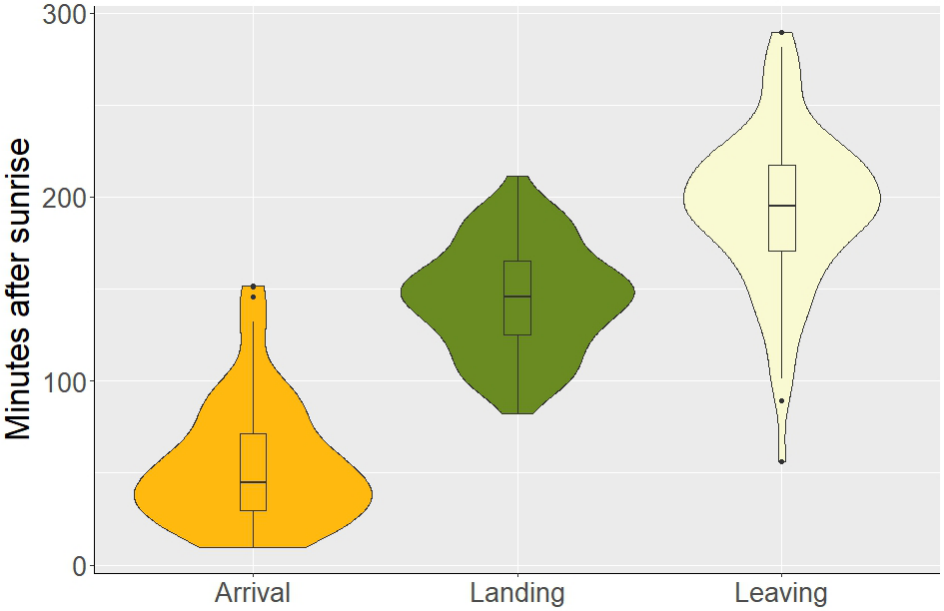
Arrival, landing and leaving times of grey parrots visiting a forest clearing in the Nkuba conservation area in the Democratic Republic of the Congo (in minutes after sunrise). The width of the violin plots shows the number of observations for each time after sunrise. The boxplot shows the median, first and third quartile.

**FIGURE 4:**
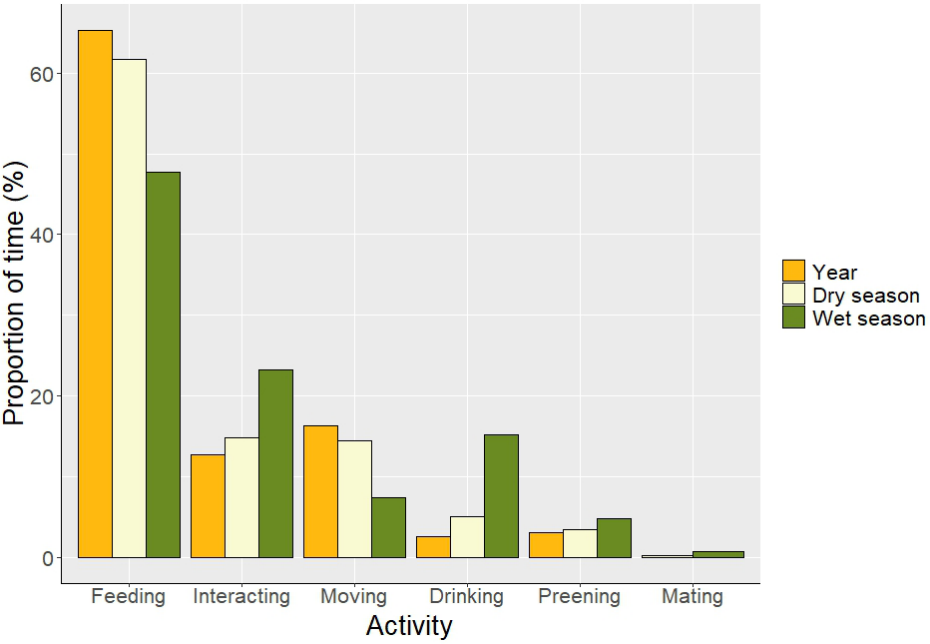
Proportion of time (%) spent by grey parrots at a forest clearing in the Nkuba conservation area in the Democratic Republic of the Congo during dry season, wet season and averaged over the entire year of the study (Year, July 2021–2022).

We found that grey parrots have a consistent pattern of visitation with distinct arrival, landing and departure times (Figure 3). On average, grey parrots started perching and vocalizing in trees surrounding the clearing 55 min after sunrise (median = 45, SD = 33, Q1 = 30, Q3 = 72, range = 10–152). Ninety minutes after initial arrival, at least one parrot would land on the ground of the clearing, quickly followed by other individuals (mean landing time = 145 min after sunrise, SD = 29, range = 82–211). Parrots stayed around 53 min in the clearing before departing (mean = 194 min after sunrise, SD = 43, range = 56–290). We found that there were on average 39.4 parrots (SD = 28.2) on the ground at any given time, though we recorded groups of up to 155 individuals. We recorded 805 focal samples, spanning a total of 57.9 h, from which we derived that grey parrots spent most of their time feeding (62%), interacting with each other (15%) and moving around (15%). They spent smaller portions of their time (<3%) drinking, preening or mating (Figure 4).

Generalized linear models revealed that during the wet season, parrots landed later (*p* = .001, *R* ^2^ = .2, see all results of the GLMs in Table 2), and remained for a shorter period of time (*p* = .003, *R* ^2^ = .2), than during the dry season. They also spent a significantly larger portion of time drinking water during the wet seasons (2.5% and 15.2% of time during the dry and wet seasons, respectively, *p* = .005, *R* ^2^ = .06, Figure 4). There were no significant differences between seasons in the proportion of time spent on other behaviours.

**TABLE 2:** Output of explanatory models for patterns in the arrival time, landing time, departure time, visit duration, presence/absence and group size of grey parrots visiting clearings in the Nkuba conservation area, with significant effects in bold.

Model	GAM				GLM											
Response	Month				Season				Morning weather				Disturbance			
	edf	*F*	*p*‐value	Highest/lowest	Estimate	Std. Error	*t*‐value	*p*‐value	Estimate	Std. Error	*t*‐value	*p*‐value	Estimate	Std. Error	*t*‐value	*p*‐value
Visit duration	3.1	15.1	**<.001**	Jul/Nov	−24.4	7.9	−3.1	**.003**	−26.2	10.3	−2.6	**.01**	−28.2	8.3	−3.4	**<.001**
Arrival time	6.7	6.2	**<.001**	Feb/Jul	−2.0	7.3	−0.3	.8	19.2	9.3	2.1	**.05**				
Landing time	2.4	6.1	**.003**	Dec/Jul	24.1	7.2	3.3	**.001**	30.7	12.7	2.4	**.02**				
Departure Time	1	4.7	**.033**	Jul/Jul	−8.8	9.6	−.09	.4	−12.4	15.2	−0.8	.4	−22.5	9.6	−2.3	**.02**
Presence/absence	2.7	5.2	**.003**	Jul/Nov	−0.4	0.6	−0.7	.5	−2.5	0.7	−3.8	**<.001**				
Group size	6.5	7.4	**<.001**	Aug/Sep	−23.1	7.7	−3.0	**.004**	−29.0	9.9	−2.9	**.004**				

*Note*: Generalized additive models (GAM), with months with highest/lowest predicted values, for arrival, landing and departure times this implies the latest/earliest times (in minutes after sunrise), respectively; Generalized linear models (GLM). Boxes in grey represent untested combinations of variables.

Additionally, we found that parrots, which landed in the clearing 77 of 100 days of observation, were more likely to land in the clearing on dry mornings rather than on rainy mornings (*p* < .001, *R* ^2^ = .2) and that on dry mornings, they arrived earlier (*p* = .05 and *R* ^2^ = .05), landed earlier (*p* = .02, *R* ^2^ = .2) and stayed longer (*p* = .01, *R* ^2^ = .2). We also found that parrots landed in larger groups during the dry season (*p* = .004 and *R* ^2^ = .2) and on dry mornings (*p* = .004 and *R* ^2^ = .2). Finally, we found that parrots visited clearings for shorter periods of time (*p* = .001, *R* ^2^ = .2) on days with at least one disturbance.

**FIGURE 5:**
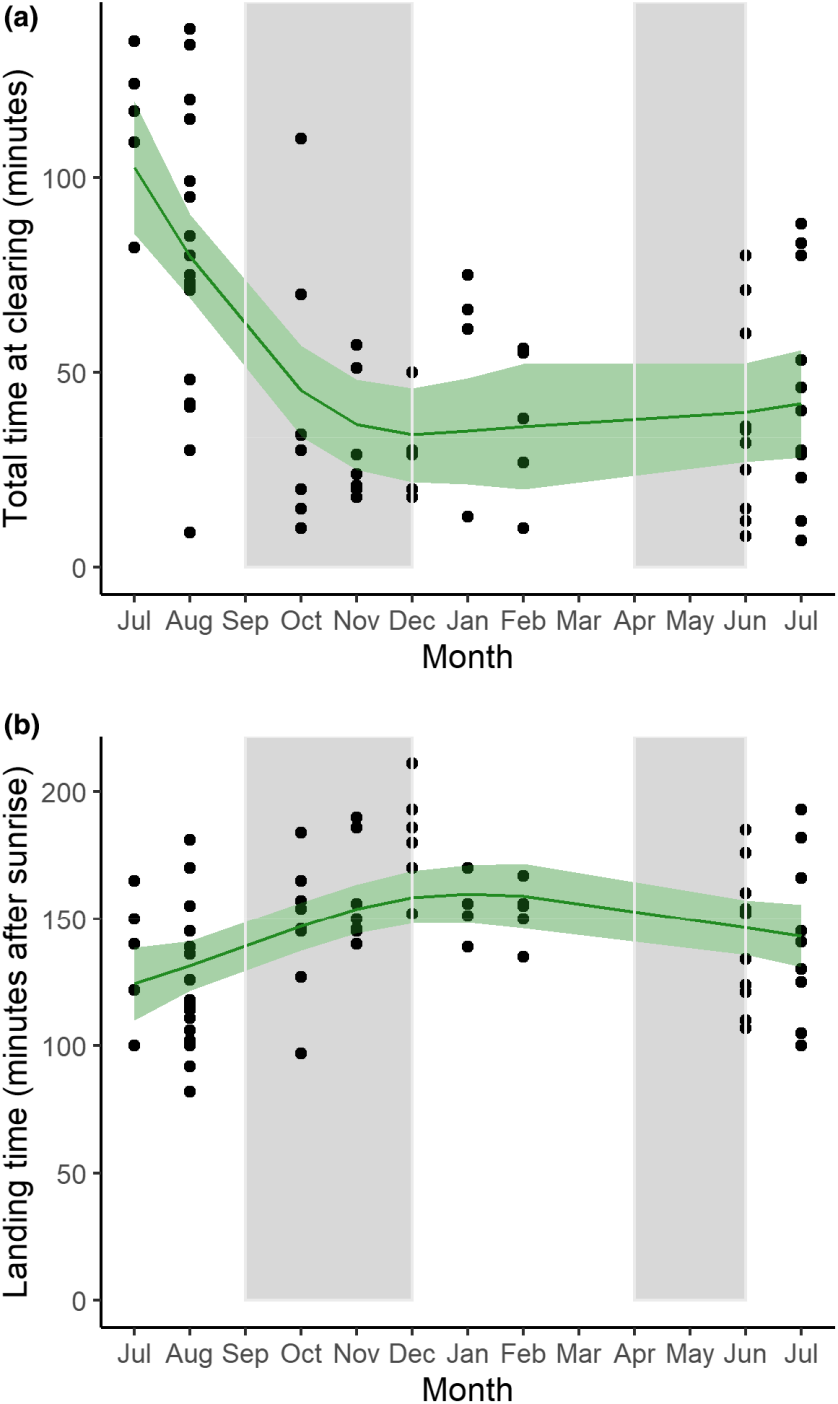
Examples of temporal (monthly) patterns in grey parrot visits of forest clearings in the Nkuba conservation area as observed between July 2021 and July 2022. Panel (a) shows that daily visits are particularly long in duration during the dry months of July–August, whereas panel (b) indicates that parrots land earlier during dry (June–August, January–March) than wet (specifically September–December, with insufficient data for April–May) season. Dots represent daily data, the smooth green lines denote trends derived from fitted generalized additive models (GAMs; light green = SE), and grey vertical columns indicate the rainy seasons.

Monthly visitation patterns corroborated our observations of seasonal patterns in clearing visitation and revealed additional, significant fluctuations for visit duration, arrival time, landing and departure time, presence/absence and group size (GAMs: *p* < .05 for all; Table 2, Figure 5). We found that daily visits were especially long in the July (dry season) and short over the September–December wet season—lack of data does not allow us to confirm if this pattern also holds for the April–May wet season—a result of early arrival and landing times and late departure times in July, and relatively later arrival and landing times in other months (February and December in particular). Similarly, the likelihood of parrots visiting on a given day (presence/absence) and group size peaked during the dry season months of July and August and were reduced over the September–December wet season.

## 4 DISCUSSION

Our study is the first to quantify the timing of grey parrot behaviours across their annual cycle. Our observations in Ungwe confirm that the clearing is heavily used by wild grey parrots throughout the year, that they visit the clearing on a near‐daily basis and that these visits occur after sunrise and end before noon. Additionally, we observed that when visiting these clearings, parrots followed a pattern consistent to those described in Bentley (1999) and May (1996): Soon after dawn, parrots gather and vocalize in the trees near the clearing. Landing occurs roughly an hour later. Grey parrots mainly feed, interact and drink at the clearing for about another hour, or until there are disturbances (e.g. human noises, presence of raptors or alarm calls emitted by the Greater Blue Turacos). Such consistent patterns and the high frequencies of the visits suggest that visitation of clearings constitutes an important component of the daily routine of the parrots and plays a key role in the ecology of these endangered birds.

We observed that grey parrots spent most of their time feeding in the clearing, supporting previous observations of similar behaviours and indicating that these clearings constitute an important food source for the species (Bentley, 1999). While grey parrots are mostly known to feed on buds, flowers, seeds and fruits of trees (Tamungang & Ajayi, 2003), we corroborate findings that they also consume ground plants when visiting waterlogged clearings (Bentley, 1999). Although we were unable to detect geophagy during our observations, grey parrots may ingest specific minerals via the consumption of (muddy) water or ground plants present in the area. Future research should focus on studying the seasonal vegetation composition and testing the composition of the water and the soil of the clearing. Additional studies on the diet of wild grey parrots might also be required to determine the exact reasons behind clearing visits.

Even though they were rather consistent, visitation patterns of the clearing fluctuated throughout the year, with peaks in the likelihood, duration and number of parrots visiting during the dry months of July and August. The larger groups we observed during the dry seasons could be driven by the need for additional food resources and/or detoxifying agents necessary to supplement a poorer and more toxic diet caused by scarcity of food in the forest (Adamescu et al., 2018; Tamungang & Ajayi, 2003; Van Der Hoek et al., 2021). This also explains why the clearing was particularly attractive to parrots during the dry months of July and August, during the beginning of the chick‐rearing period when all available food sources must be exploited (Brightsmith et al., 2018). Yet, the main predictor of the presence of parrots in the clearing across the year was rainfall, with parrots landing less often in the clearing when it rained. Rain is indeed associated with diminished bird activity and was shown to prevent parrots from visiting licks in Peru (Brightsmith, 2004).

We also found that during the dry season, when flocks are bigger, parrots landed earlier and stayed longer, a pattern possibly linked to anti‐predator defence behaviour (Burger & Gochfeld, 2003). Main predators of grey parrots are large cats, raptors (e.g. goshawks regularly observed flying in the clearing, Table A1), and humans (Piebeng et al., 2018), to which Grey parrots are particularly vulnerable when they feed on the treeless ground of the clearing. Larger flocks are thus better protected against these threats as it allows the group to have more individuals watching out for predators while parrots are feeding (Westcott et al., 1988) and large flocks taking off together tend to confuse predators, diminishing the risk for a successful attack (Landeau & Terborgh, 1986). When parrots were disturbed and took off, they generally all left the area and did not return to the clearing, which is consistent with findings from Dzangha NP (May, 1996).

Unfortunately, we were unable to visit the clearing during the short rainy season of April to May. This can impact our results as we may have missed some temporal variations within the annual cycle, especially since this period corresponds to the beginning of the breeding season of the parrots, when they court each other, search for nests in tree cavities and lay eggs (Piebeng et al., 2018). It is difficult to know whether parrots would vary their visitation patterns of the clearing during this period. While our data during the wet season of October to December show that parrots are less likely to visit the clearing on rainy mornings, which occur more often during the rainy season, our results also suggest that parrots visit the clearing more often in July and August, during the peak of the breeding season. It is therefore important that future research focuses on gathering more data on the use of clearings during the beginning of the breeding season. More observations during this period, during which parrots court each other and mate, might also increase the number of mating activities observed in the clearing, since parrots do not mate near their nests (Piebeng et al., 2018).

In Ungwe, we observed groups of up to 155 parrots visiting the clearings, though this is an underestimation of the total number of parrots in the area as we could only count the parrots present on the ground (Bentley, 1999). Nevertheless, considering the spectacular groups of hundreds of parrots reported to visit the Lomami NP located a few hundred kilometres from the NCA (Hart, 2013), groups of over 800 individuals in Cameroon (Bentley, 1999), and roosting sites with 700–3000 grey parrots in Ghana (Annorbah et al., 2015), we may interpret our numbers as relatively low. Since there are natural local variations in food availability and in the densities of parrots within their range (Marsden et al., 2016), it is possible that the small group size we observed is in line with historical numbers in the area. However, capture for the pet trade recently occurred ‐ and may still be occurring – in and near the NCA (Personal observations), so grey parrots in this region may have experienced declines due to harvesting for the pet trade (Hart, 2013; Hart et al., 2016).

While our research shows the importance of the Ungwe clearing as a food source for grey parrots across an annual cycle, there may be variation in visitation patterns between clearings and years. Yet, the visitation patterns and activities carried out by grey parrots we observed, in a clearing located in the easternmost part of their range, were consistent with observations made in clearings in Southern Cameroon (Bentley, 1999) and Central African Republic (May, 1996), in the western part of their range. We can therefore assume that visitation patterns are likely similar in all clearings visited by grey parrots throughout their range. Likewise, we assume that feeding is the most important activity carried out in the clearings. However, since we found that the season influences how long parrots stay in the clearing and that parrots spent more time drinking during the rainy season, it is likely that inter‐annual climatic variations would affect visitation patterns and the time parrots spend drinking or eating at the clearing. Additionally, changes in rainfall patterns caused by climate change (Brawn et al., 2017) would also affect food and water availability in the forest, and therefore, the importance of the clearing as a food and drinking source for the parrots. This could, in turn, potentially lead to a change in visitation patterns of the clearing and in the activities grey parrots perform there.

Given the importance of clearing visits for parrots and other species, we suggest that conservation actions focus on reducing anthropogenic disturbances in and around the clearing in the critical hours after sunrise. As visitation patterns appears to be consistent across the few similar clearings observed, we recommend that this strategy is adopted throughout the species' range. Needless to say, these conservation actions of grey parrots should involve local communities as key actors of conservation (Dawson et al., 2021). Increasing awareness about the need to protect grey parrots should be a priority as members of the local communities can help identifying – and stopping – illegal poaching activities carried out in the area. Additionally, the Dian Fossey Fund and local communities can work together to make sure that communities have alternative incomes to alleviate the need to rely on capture and trade of endangered species like grey parrots (Hackel, 1999; Martin et al., 2014).

## AUTHOR CONTRIBUTIONS

**C. Fastré:** Conceptualization (lead); data curation (lead); formal analysis (lead); methodology (lead); writing – original draft (lead). **B. Igwili:** Data curation (equal); investigation (lead); methodology (supporting). **F. Van de Perre:** Conceptualization (lead); formal analysis (supporting); methodology (lead); writing – original draft (supporting); writing – review and editing (equal). **Y. van der Hoek:** Conceptualization (supporting); formal analysis (supporting); writing – review and editing (equal).

## CONFLICT OF INTEREST STATEMENT

The authors declare that they have no conflicts of interests.

## Data Availability

All data is available at: Fastre, Constance; Van de Perre, Frederik; van der Hoek, Yntze (2023), ‘Behavioural patterns of endangered grey parrots (*Psittacus erithacus*) in a forest clearing in the Democratic Republic of the Congo’, Mendeley Data, V1, doi: 10.17632/2ywnxjz2mr.1

## APPENDIX A

**TABLE A1:** Bird and mammal species observed in the clearing of Ungwe in the Nkuba conservation area during the study period. Bird taxonomy followed (HBW and BirdLife International, 2022).

Species name	English name	Heard	Seen
Mammals			
*Cercopithecus hamlyni*	Owl‐faced Monkey	×	×
*Gorilla beringei graueri*	Eastern Lowland Gorilla		× (camera trap)
*Syncerus caffer nanus*	African Forest Buffalo		× (camera trap)
Birds			
Galliformes			
*Numida* sp.	Guineafowl sp.	×	
*Sarothrura pulchra*	White‐spotted Flufftail	×	
Anseriformes			
Pteronetta hartlaubii	Hartlaub's Duck		×
Apodiformes			
*Apus apus*	Common Swift		×
Musophagiformes			
*Corythaeola cristata*	Great blue Turaco	×	×
*Tauraco schuettii*	Black‐billed Turaco	×	×
Cuculiformes			
*Cercococcyx mechowi*	Dusky Long‐tailed Cuckoo	×	
*Cercococcyx olivinus*	Olive Long‐tailed Cuckoo	×	
*Chrysococcyx cupreus*	African Emerald Cuckoo	×	×
*Cuculus clamosus*	Black Cuckoo	×	
*Cuculus solitarius*	Red‐Chested Cuckoo	×	
Columbiformes			
*Columba iriditorques*	Western Bronze‐naped Pigeon	×	
*Columba unicincta*	Afep Pigeon	×	×
*Streptopelia semitorquata*	Red‐eyed Dove	×	×
*Treron calvus*	African Green Pigeon	×	×
*Turtur afer*	Blue‐spotted Wood‐dove	×	
*Turtur brehmeri*	Blue‐headed Wood‐dove	×	
*Turtur tympanistria*	Tambourine Dove	×	
Charadriiformes			
*Actitis hypoleucos*	Common Sandpiper		×
Ciconiiformes			
*Scopus umbretta*	Hamerkop	×	
Accipitiformes			
*Accipiter tachiro*	African Goshawk	×	×
*Buteo buteo*	Common Buzzard	×	
Trogoniformes			
*Apaloderma narina*	Narina Trogon	×	
Bucerotiformes			
*Bycanistes albotibialis*	White‐thighed Hornbill	×	×
*Bycanistes sharpii*	Eastern piping Hornbill	×	×
*Bycanistes subcylindricus*	Black‐and‐white Casqued Hornbill	×	×
*Ceratogymna atrata*	Black‐casqued Hornbill	×	×
*Horizocerus albocristatus*	White‐crested Hornbill	×	×
*Lophocerus camurus*	Red‐billed Dwarf Hornbill	×	×
*Lophocerus fasciatus*	Congo Pied Hornbill	×	×
Coraciiformes			
*Alcedo quadribrachys*	Shining‐Blue Kingfisher	×	
*Halcyon badia*	Chocolate‐Backed Kingfisher	×	×
*Halcyon leucocephala*	Grey‐headed kingfisher	×	×
*Halcyon malimbica*	Blue‐breasted Kingfisher	×	×
*Halcyon senegalensis*	Woodland Kingfisher	×	×
*Merops apiaster*	European Bee‐eater	×	×
*Merops gularis*	Black Bee‐eater	×	×
Piciformes			
*Dendropicos* sp.		×	
*Verreauxia africana*	African Piculet	×	×
Psittaciformes			
*Psittacus erithacus*	Grey parrot	×	×
Passeriformes			
*Alethe castanea*	Fire‐crested Alethe	×	
*Andropadus latirostris*	Yellow‐whiskered Greenbul	×	×
*Apalis cinerea*	Grey Apalis	×	×
*Baeopogon indicator*	Honeyguide Greenbul		×
*Bleda* sp.		×	
*Buccanodon duchaillui*	Yellow‐spotted Barbet	×	
*Camaroptera chloronota*	Olive‐green Camaroptera	×	×
*Centropus grillii*	Black Coucal	×	
*Centropus monachus*	Blue‐headed Coucal	×	×
*Centropus senegalensis*	Senegal Coucal	×	
*Cinnyris chloropygius*	Olive‐bellied Sunbird	×	×
*Criniger calurus*	Red‐tailed Greenbul	×	
*Criniger chloronotus*	Eastern Bearded Greenbul	×	
*Cyanomitra olivacea*	Olive Sunbird	×	×
*Dicrurus* sp.		×	
*Eurillas ansorgei*	Ansorge's Greenbull	×	
*Eurillas virens*	Little Greenbull	×	×
*Gymnobucco sladeni*	Sladen's Barbet	×	
*Hylia prasina*	Green Hylia	×	×
*Illadopsis albipectus*	Scaly‐breasted Illadopsis	×	
*Illadopsis fulvescens*	Brown Illadopsis	×	
*Illadopsis rufipennis*	Pale‐breasted Illadopsis	×	×
*Ixonotus guttatus*	Spotted Greenbull	×	×
*Malimbus cassini*	Cassin's Malimbe	×	
*Malimbus malimbicus*	Crested Malimbe	×	
*Malimbus nitens*	Blue‐billed Malimbe	×	×
*Neocossyphus poensis*	White‐tailed Ant‐thrush	×	×
*Neocossyphus rufus*	Red‐tailed Ant‐thrush	×	
*Nicator chloris*	Western Nicator	×	
*Nigrita luteifrons*	Pale‐fronted Nigrita	×	×
*Oriolus brachyrhynchus*	Western Black‐headed Oriole	×	
*Phyllastrephus xavieri*	Xavier's Greenbul	×	
*Pogoniulus atroflavus*	Red‐rumped Tinkerbird	×	
*Pogoniulus bilineatus*	Yellow‐rumped Tinkerbird	×	×
*Pogoniulus scolopaceus*	Speckled Tinkerbird	×	
*Pogoniulus subsulphureus*	Yellow‐throated Tinkerbird	×	
*Smithornis rufolateralis*	Rufous‐sided Broadbill	×	
*Stizorhina fraseri*	Rufous Flycatcher‐thrush	×	
*Terpsiphone batesi*	Bates's Paradise Flycatcher	×	
*Terpsiphone bedfordi*	Bedford's Paradise Flycatcher	×	
*Terpsiphone rufiventer*	Red‐bellied Paradise Flycatcher	×	×

**TABLE A2:** Sunrise hours (HH:MM:SS) for the days (D/M/Y) of observations of the grey Parrots in Ungwe (CAT, Central Africa Time).

Date	Sunrise	Date	Time	Date	Time
26/07/2021	06:14:30	11/11/2021	05:48:43	16/06/2022	06:08:54
28/07/2021	06:14:27	12/11/2021	05:48:49	17/06/2022	06:09:07
29/07/2021	06:14:24	13/11/2021	05:48:55	18/06/2022	06:09:20
30/07/2021	06:14:21	14/11/2021	05:49:02	19/06/2022	06:09:33
31/07/2021	06:14:17	15/11/2021	05:49:10	20/06/2022	06:09:46
01/08/2021	06:14:12	16/11/2021	05:49:19	21/06/2022	06:09:59
02/08/2021	06:14:07	06/12/2021	05:55:08	22/06/2022	06:10:12
03/08/2021	06:14:01	07/12/2021	05:55:33	23/06/2022	06:10:25
04/08/2021	06:13:55	08/12/2021	05:55:59	24/06/2022	06:10:38
05/08/2021	06:13:48	09/12/2021	05:56:24	25/06/2022	06:10:51
06/08/2021	06:13:40	10/12/2021	05:56:51	26/06/2022	06:11:03
21/08/2021	06:10:35	11/12/2021	05:57:18	13/07/2022	06:13:53
22/08/2021	06:10:18	12/12/2021	05:57:45	14/07/2022	06:13:59
23/08/2021	06:10:01	13/12/2021	05:58:12	15/07/2022	06:14:05
24/08/2021	06:09:44	14/12/2021	05:58:40	16/07/2022	06:14:10
25/08/2021	06:09:26	15/12/2021	05:59:09	17/07/2022	06:14:15
26/08/2021	06:09:08	16/12/2021	05:59:37	18/07/2022	06:14:19
27/08/2021	06:08:49	17/12/2021	06:00:06	19/07/2022	06:14:22
28/08/2021	06:08:30	14/01/2022	06:13:14	20/07/2022	06:14:25
29/08/2021	06:08:10	15/01/2022	06:13:37	21/07/2022	06:14:28
30/08/2021	06:07:50	16/01/2022	06:13:59	22/07/2022	06:14:30
31/08/2021	06:07:30	17/01/2022	06:14:21	23/07/2022	06:14:31
02/10/2021	05:55:31	18/01/2022	06:14:42	24/07/2022	06:14:31
03/10/2021	05:55:10	19/01/2022	06:15:02		
04/10/2021	05:54:49	20/01/2022	06:15:22		
06/10/2021	05:54:08	21/01/2022	06:15:41		
07/10/2021	05:53:49	22/01/2022	06:15:59		
10/10/2021	05:52:52	23/01/2022	06:16:17		
11/10/2021	05:52:34	24/01/2022	06:16:33		
12/10/2021	05:52:17	25/01/2022	06:16:49		
13/10/2021	05:52:00	04/02/2022	06:18:47		
05/11/2021	05:48:29	05/02/2022	06:18:54		
06/11/2021	05:48:29	06/02/2022	06:19:00		
07/11/2021	05:48:30	08/02/2022	06:19:11		
08/11/2021	05:48:32	09/02/2022	06:19:15		
09/11/2021	05:48:35	10/02/2022	06:19:19		
10/11/2021	05:48:39	15/06/2022	06:08:41		

## References

[ece370039-bib-0001] Adamescu, G. S. , Plumptre, A. J. , Abernethy, K. A. , Polansky, L. , Bush, E. R. , Chapman, C. A. , Shoo, L. P. , Fayolle, A. , Janmaat, K. R. L. , Robbins, M. M. , Ndangalasi, H. J. , Cordeiro, N. J. , Gilby, I. C. , Wittig, R. M. , Breuer, T. , Hockemba, M. B. N. , Sanz, C. M. , Morgan, D. B. , Pusey, A. E. , … Beale, C. M. (2018). Annual cycles are the most common reproductive strategy in African tropical tree communities. Biotropica, 50(3), 418–430. 10.1111/btp.12561

[ece370039-bib-0002] Akoglu, H. (2018). User's guide to correlation coefficients. Turkish Journal of Emergency Medicine, 18(3), 91–93. 10.1016/j.tjem.2018.08.001 30191186 PMC6107969

[ece370039-bib-0003] Amuno, J. B. , Massa, R. , & Dranzoa, C. (2007). Abundance, movements and habitat use by African grey parrots (*Psittacus erithacus*) in Budongo and Mabira forest reserves, Uganda. Ostrich, 78(2), 225–231. 10.2989/OSTRICH.2007.78.2.17.97

[ece370039-bib-0600] Amuno, J. B. , Massa, R. , & Okethowengo, G. (2010). Some observations on nesting African Grey Parrots, Psittacus erithacus, in Uganda. Rivista Italiana di Ornitologia, 80(1). doi: 10.4081/rio.2010.86

[ece370039-bib-0004] Annorbah, N. N. D. , Collar, N. J. , & Marsden, S. J. (2015). Trade and habitat change virtually eliminate the grey parrot *Psittacus erithacus* from Ghana. http://trade.cites.org/

[ece370039-bib-0005] Bentley, C. S. (1999). Soil‐eating by grey parrots in Cameroon: An answer to mineral deficiencies or toxins in the diet? University of Arizona.

[ece370039-bib-0006] BirdLife International . (2022). Species factsheet: Psittacus erithacus . http://www.birdlife.org.on

[ece370039-bib-0007] Blanco, G. , Hiraldo, F. , & Tella, J. L. (2018). Ecological functions of parrots: An integrative perspective from plant life cycle to ecosystem functioning. Emu, 118(1), 36–49. 10.1080/01584197.2017.1387031

[ece370039-bib-0008] Brawn, J. D. , Benson, T. J. , Stager, M. , Sly, N. D. , & Tarwater, C. E. (2017). Impacts of changing rainfall regime on the demography of tropical birds. Nature Climate Change, 7(2), 133–136. 10.1038/nclimate3183

[ece370039-bib-0009] Brightsmith, D. J. (2004). Effects of weather on parrot geophagy in Tambopata, Peru. Wilson Bulletin, 116(2), 134–145. 10.1676/03-087B

[ece370039-bib-0010] Brightsmith, D. J. , Hobson, E. A. , & Martinez, G. (2018). Food availability and breeding season as predictors of geophagy in Amazonian parrots. Ibis, 160(1), 112–129. 10.1111/ibi.12515

[ece370039-bib-0011] Brightsmith, D. J. , & Villalobos, E. M. (2011). Parrot behavior at a Peruvian clay lick. Wilson Journal of Ornithology, 123(3), 595–602. 10.1676/09-109.1

[ece370039-bib-0012] Burger, J. , & Gochfeld, M. (2003). Parrot behavior at a Rio Manu (Peru) clay lick: Temporal patterns, associations, and antipredator responses. Acta Ethologica, 6(1), 23–34. 10.1007/s10211-003-0080-y

[ece370039-bib-0013] Chalmer, B. J. (1987). Understanding statistics. Taylor & Francis. 10.4324/9780367813161

[ece370039-bib-0014] Chapman, C. A. , Chapman, L. J. , & Wrangham, R. (1993). Observations on the feeding biology and population ecology of the grey parrot *Psittacus erithacus* . Scopus, 16, 89–93.

[ece370039-bib-0015] Chupezi, T. J. , Ndoye, O. , & Mpele, T. O. (2006). Commodity‐chain analysis for the capture and trade in the African grey parrots (*Psittacus erithacus erithacus*) in Cameroon. Prepared for policy and distributional equity in natural resource commodity markets: Commodity chain analysis as a policy tool project.

[ece370039-bib-0016] Dawson, N. M. , Coolsaet, B. , Sterling, E. J. , Loveridge, R. , Gross‐Camp, N. D. , Wongbusarakum, S. , Sangha, K. K. , Scherl, L. M. , Phan, H. P. , Zafra‐Calvo, N. , Lavey, W. G. , Byakagaba, P. , Idrobo, C. J. , Chenet, A. , Bennett, N. J. , Mansourian, S. , & Rosado‐May, F. J. (2021). The role of indigenous peoples and local communities in effective and equitable conservation. Ecology and Society, 26(3), 19. 10.5751/ES-12625-260319

[ece370039-bib-0017] Dueker, S. , Kupsch, D. , Bobo, S. K. , Heymann, E. W. , & Waltert, M. (2020). Congo grey parrot *Psittacus erithacus* densities in oil palm plantation, agroforestry mosaic and protected forest in Southwest Cameroon. Bird Conservation International, 30(1), 156–167. 10.1017/S0959270919000194

[ece370039-bib-0018] Hackel, J. D. (1999). Community conservation and the future of Africa's wildlife. Conservation biology, 13(4), 726–734.

[ece370039-bib-0019] Hart, J. (2013). Congo's quintessential parrot. PittaScene, Winter, 16–19.

[ece370039-bib-0020] Hart, J. , Hart, T. , Salumu, L. , Bernard, A. , & Martin, R. (2016). Increasing exploitation of grey parrots in eastern DRC drives population declines. Oryx, 50(1), 16–17. 10.1017/s003060531500085x

[ece370039-bib-0021] HBW and BirdLife International . (2022). Handbook of the birds of the world and BirdLife international digital checklist of the birds of the world. Version 7. http://datazone.birdlife.org/userfiles/file/species/taxonomy/hbw‐birdlife_checklist_v7_dec22.zip

[ece370039-bib-0022] IUCN . (2023, January 26). *Psittacus erithacus. The* IUCN red list of threatened species 2021: e.T22724813A154428817.

[ece370039-bib-0023] Karger, D. N. , Conrad, O. , Böhner, J. , Kawohl, T. , Kreft, H. , Soria‐Auza, R. W. , Zimmermann, N. E. , Linder, H. P. , & Kessler, M. (2017). Climatologies at high resolution for the earth's land surface areas. Scientific Data, 4, 170122. 10.1038/sdata.2017.122 28872642 PMC5584396

[ece370039-bib-0024] Landeau, L. , & Terborgh, J. (1986). Oddity and the “confusion effect” in predation. Animal Behaviour, 34, 1372–1380.

[ece370039-bib-0025] Marsden, S. J. , Loqueh, E. , Takuo, J. M. , Hart, J. A. , Abani, R. , Ahon, D. B. , Annorbah, N. N. D. , Johnson, R. , & Valle, S. (2016). Using encounter rates as surrogates for density estimates makes monitoring of heavily‐traded grey parrots achievable across Africa. Oryx, 50(4), 617–625. 10.1017/S0030605315000484

[ece370039-bib-0026] Martin, R. O. , Perrin, M. R. , Boyes, R. S. , Abebe, Y. D. , Annorbah, N. D. , Asamoah, A. , Bizimana, D. , Bobo, K. S. , Bunbury, N. , Brouwer, J. , Diop, M. S. , Ewnetu, M. , Fotso, R. C. , Garteh, J. , Hall, P. , Holbech, L. H. , Madindou, I. R. , Maisels, F. , Mokoko, J. , … Wondafrash, M. (2014). Research and conservation of the larger parrots of Africa and Madagascar: A review of knowledge gaps and opportunities. Ostrich, 85(3), 205–233. 10.2989/00306525.2014.948943

[ece370039-bib-0700] Martin, P. & Bateson, P. (2007). Measuring behaviour: An introductory guide Third Edition. Cambridge University Press.

[ece370039-bib-0500] Martin, R. O. (2018). The wild bird trade and African parrots: Past, present and future challenges. Ostrich, 1–5. doi: 10.2989/00306525.2017.1397787

[ece370039-bib-0027] May, D. (1996). The behavior of African grey parrots om the rainforest of the Central African Republic. Psittascene, 8, 8–9.

[ece370039-bib-0028] May, D. (2001). Grey parrots of The Congo Basin Forest. PsittaScene, 13(2), 8–10.

[ece370039-bib-0029] Navarro, D. J. (2015). Learning statistics with R: A tutorial for psychology students and other beginners (0.6). University of South Wales.

[ece370039-bib-0030] Piebeng, G. N. K. , Tamungang, S. A. , & Teguia, A. (2018). Breeding biology of African grey parrot (*Psittacus erithacus*) in Kom National Park (South‐Cameroon) and implications to the species conservation. International Journal of Biological and Chemical Sciences, 11(5), 1948. 10.4314/ijbcs.v11i5.2

[ece370039-bib-0031] R Core Team . (2022). R: A language and environment for statistical computing. R Foundation for Statistical Computing. https://www.R‐project.org/

[ece370039-bib-0032] Ripley, B. , Venables, B. , Bates, D. , Hornik, K. , Gebhardt, A. , & Firth, D. (2013). Package “mass” .

[ece370039-bib-0033] Tamungang, S. A. , & Ajayi, S. S. (2003). Diversity of food of the Grey parrot Psittacus erithacus in Korup National Park, Cameroon. Bulletin of the African Bird Club, 10(1), 33–36. 10.5962/p.309683

[ece370039-bib-0034] Tamungang, S. A. , Cheke, R. A. , Mofor, G. Z. , Tamungang, R. N. , & Oben, F. T. (2014). Conservation concern for the deteriorating geographical range of the grey parrot in Cameroon. International Journal of Ecology, 2014, 753294. 10.1155/2014/753294

[ece370039-bib-0035] Tamungang, S. A. , Onabid, M. A. , Awa, T. , & Balinga, V. S. (2016). Habitat preferences of the grey parrot in heterogeneous vegetation landscapes and their conservation implications. International Journal of Biodiversity, 2016, 1–10. 10.1155/2016/7287563

[ece370039-bib-0036] Van Der Hoek, Y. , Pazo, W. D. , Binyinyi, E. , Ngobobo, U. , Stoinski, T. S. , & Caillaud, D. (2021). Diet of Grauer's gorillas (*Gorilla beringei graueri*) in a low‐elevation Forest. Folia Primatologica, 92(2), 126–138. 10.1159/000515377 33882499

[ece370039-bib-0037] van Rij, J. , Wieling, M. , Baayen, R. , & Van Rijn, H. (2020). itsadug: Interpreting time series and autocorrelated data using GAMMs . R Package Version 2.4.

[ece370039-bib-0038] Westcott, A. B. , David, A. , & Cockburn, A. (1988). Flock size and vigilance in parrots. In. Australian Journal of Zoology, 36, 335.

[ece370039-bib-0039] Wickham, H. , Averick, M. , Bryan, J. , Chang, W. , McGowan, L. , François, R. , Grolemund, G. , Hayes, A. , Henry, L. , Hester, J. , Kuhn, M. , Pedersen, T. , Miller, E. , Bache, S. , Müller, K. , Ooms, J. , Robinson, D. , Seidel, D. , Spinu, V. , … Yutani, H. (2019). Welcome to the Tidyverse. Journal of Open Source Software, 4(43), 1686. 10.21105/joss.01686

[ece370039-bib-0040] Wood, S. , & Wood, M. (2015). Package “mgcv.” .

